# The Pivotal Role of Noncoding RNAs in Flowering Time Regulation

**DOI:** 10.3390/genes14122114

**Published:** 2023-11-23

**Authors:** Yun Liu, Qing-Feng Zhu, Wen-Yan Li, Pei Chen, Jiao Xue, Yang Yu, Yan-Zhao Feng

**Affiliations:** Guangdong Key Laboratory of Crop Germplasm Resources Preservation and Utilization, Agro-biological Gene Research Center, Guangdong Academy of Agricultural Sciences, Guangzhou 510640, China; vincent123ly@163.com (Y.L.); zhuqingfeng@gdaas.cn (Q.-F.Z.); liwy1023@foxmail.com (W.-Y.L.); chenpei@gdaas.cn (P.C.); xuejiao@gdaas.cn (J.X.)

**Keywords:** noncoding RNAs, flowering time, biological role, molecular mechanism

## Abstract

Noncoding RNAs constitute a substantial portion of the transcriptome and play pivotal roles in plant growth and development. Among these processes, flowering stands out as a crucial trait, ensuring reproductive success and seed set, and is meticulously controlled by genetic and environmental factors. With remarkable advancements in the identification and characterization of noncoding RNAs in plants, it has become evident that noncoding RNAs are intricately linked to the regulation of flowering time. In this article, we present an overview of the classification of plant noncoding RNAs and delve into their functions in the regulation of flowering time. Furthermore, we review their molecular mechanisms and their involvement in flowering pathways. Our comprehensive review enhances the understanding of how noncoding RNAs contribute to the regulation of flowering time and sheds light on their potential implications in crop breeding.

## 1. Introduction

For angiosperms, the transition from vegetative growth to reproductive growth is marked by floral induction, a critical stage in their growth cycle. As plants mature, they undergo a shift from vegetative growth to reproductive growth, taking advantage of suitable conditions such as the right seasons, temperatures, and light exposure to yield high-quality, fertile seeds. The regulation of flowering time in plants holds paramount significance, not only for ensuring the continuity of plant populations but also for profoundly influencing attributes like the plant’s reproductive span, seed yield, quality, and their relevance to social production.

The process of flowering in plants is intricately governed by a complex interplay of factors, encompassing external elements and internal signals [1]. Extensive research on model plants such as *Arabidopsis thaliana* and *Oryza sativa* has unveiled four primary pathways governing flowering in plants: the photoperiodic pathway, vernalization and the autonomous pathway, the age pathway, and gibberellin acid (GA) regulation. Additionally, signals like circadian rhythms, sugars, and brassinosteroids participate in orchestrating the transition to the flowering phase. These internal signals and external cues predominantly converge through a substance known as florigen, a compound of stimuli that moves from the leaves to the shoot apical meristem to induce flowering encoded by *FLOWERING LOCUS T* (*FT*) [2], orchestrating the expression of genes related to meristem differentiation and stimulating the development of reproductive structures in the shoot apical meristem (SAM).

Notably, it has come to light that in the eukaryotic genome, a mere 2% of transcripts translate into peptides, with most lacking protein-coding capacity and falling under the category of noncoding RNAs [3]. Beyond structural RNAs like tRNAs, rRNAs, snRNAs, and snoRNAs, other noncoding RNAs were once regarded as inert “junk DNAs” until meticulous research substantiated their functionality [4]. Over the past two decades, numerous studies have underscored the indispensable role of noncoding RNAs in various aspects of a plant’s lifecycle, encompassing seed germination [5], root and leaf development [6,7], floral transition [8], pollen development [9], fruit ripening [10], and grain yield [11,12]. Noncoding RNAs also partake in the management of stress responses [13]. Compared to protein-coding genes, noncoding RNAs gently modulate biological processes by influencing the quantity rather than the quality of downstream targets. This characteristic makes noncoding RNAs invaluable tools for fine-tuning gene expression and traits.

The past summaries of flowering time studies have predominantly focused on protein-coding genes. However, noncoding RNAs play a crucial role in flowering regulation, yet there has been limited comprehensive summarization in this area. In line with the advancements in next-generation sequencing and experimental techniques, an increasing array of noncoding RNAs is being identified as key players in the regulation of flowering time. Scientists are leveraging noncoding RNAs to intricately adjust the timing of flowering, benefiting crop production [14]. Here, we will summarize noncoding RNAs in regulating flowering time according to their involved pathways, discuss the variation of noncoding RNAs in adaption to flowering time, and highlight several unresolved questions in this field.

## 2. Classification of Noncoding RNAs and Their General Mechanisms

Noncoding RNAs are categorized based on their length, which gives rise to three primary groups: small RNAs (<50 nt), intermediate-sized noncoding RNAs (50–200 nt), and long noncoding RNAs (lncRNAs, >200 nt) [15]. Because intermediate-sized ncRNA primarily comprises snoRNA and snRNA, responsible for tRNA and snRNA modifications and splicing, specific member function studies remain limited [16,17]. Hence, this paper primarily focuses on the roles of small RNA and lncRNA in flowering. 

In terms of their origins, small RNAs can be further divided into two prominent subtypes: microRNAs (miRNAs) and small interfering RNAs (siRNAs). The transcription of miRNAs is conducted by DNA-dependent RNA polymerase II (Pol II) from MIRNA loci. The initial transcript, known as pri-miRNA, is characterized by an m7G cap and polyadenylation, and it forms a distinctive hairpin structure [18,19]. This structure is then recognized and cleaved by Dicer-like 1 (DCL1), leading to the formation of an miRNA/miRNA* duplex. The mature miRNA is subsequently loaded onto AGO1, giving rise to the RNA-induced silencing complex (RISC), while the miRNA* is degraded [20,21]. MiRNAs primarily act through complementary binding to target mRNA, leading to mRNA cleavage. In some cases, miRNAs can also reduce protein abundance at the translational level. For instance, miR156/miR157 cleave *SPLs* transcripts and inhibit their translation, albeit with varying preference for different targets [22]. Furthermore, miR172 predominantly represses the translation of *AP2* and induces the cleavage of *AP2* mRNA [23]. 

PhasiRNAs, also known as phased small interfering RNAs, are secondary siRNAs generated from extended RNA precursors. These precursor RNAs are cleaved by an miRNA (referred to as the trigger), and the second strand is synthesized by RNA-dependent RNA polymerase 6 (RDR6). DCL4/5 process the double-stranded RNA into segments measuring 21/24 nt, arranged in an array and featuring a 2 nt overhang at the 5′ end of each strand [24]. PhasiRNAs operate similarly to miRNAs, binding to target mRNAs and causing mRNA cleavage or DNA methylation. The 24 nt phasiRNAs predominantly occur during meiosis and are crucial for male fertility in plants. Research has highlighted a strong association between 24 nt phasiRNAs and the highly methylated CHH DNA at PHAS loci [25], exerting transcriptional control over gene expression, which differs from the cleavage-based regulation observed with 21 nt phasiRNAs. PhasiRNAs play a role in amplifying the silencing signals, thereby further promoting the degradation of target genes. For instance, in citrus, the phasiRNA Cs1g09635 3′D6(-) cleaves *NAC* transcripts, leading to the downregulation of target genes alongside the trigger miRNA [26]. 

LncRNAs are primarily transcribed by Pol II, IV, and V [27]. They can originate from either the sense or antisense strand of an exon, intron, or intergenic region. LncRNAs can function as guides, associating with proteins or scaffolds that bind to DNA or RNA. In this capacity, they epigenetically regulate gene expression. LncRNAs can also serve as decoys, sequestering small RNAs and attenuating their functions. For example, *COOLAIR* regulates chromatin states and demethylates H3K4me2 by interacting with the *FLOWERING LOCUS C (FLC)* locus [28], which encodes a MADS-box transcription factor that acts as a flowering repressor. Conversely, *COLDAIR* recruits the Polycomb Repressive Complex 2 (PRC2) and enhances H3K27me3 modifications on *FLC*, repressing its transcription [29]. Apart from binding DNA in a cis-acting manner, lncRNAs can also target DNA in a trans-acting fashion. A recent study unveiled *FLAIL* as a trans-acting RNA that interacts with specific flowering-repressor genes to regulate their alternative splicing [30].

Circular RNAs are a novel class of closed noncoding RNAs that are formed through covalent bonding between the downstream 3′ splice site and the upstream 5′ splice site. CircRNAs may be derived as by-products of mRNA splicing, from noncoding RNAs by adenosine deaminase, or through non-canonical alternative splicing events [31,32,33]. The closed structure, devoid of exposed 5′ and 3′ ends, renders circRNAs insensitive to RNase R, facilitating their enrichment and purification from numerous linear RNAs. Recent studies have found that circRNAs in plants can regulate the number of petals and stamens [34], enhance rice resistance to blast disease [35], and confer resistance to abiotic stresses such as cold and drought [36,37]. CircRNAs can function as miRNA decoys, indirectly enhancing the expression of miRNA target genes. For example, soybean circ-CCR2 acts as a sponge for miR172, while circ-SEC5A and circ-EF1B serve as sponges for miR156. Collectively, they mediate the expression of regulators involved in the transition to the floral fate [38]. Additionally, the lariat RNA derived from *At5g37720*, which is a lariat-formed intron generated by mRNA splicing, suppresses *FT* expression through an unknown mechanism [39]. As in-depth studies on plant circRNAs continue to evolve, we can expect to gain a clearer understanding of the intricate mechanical aspects of circRNA function.

**Figure 1:**
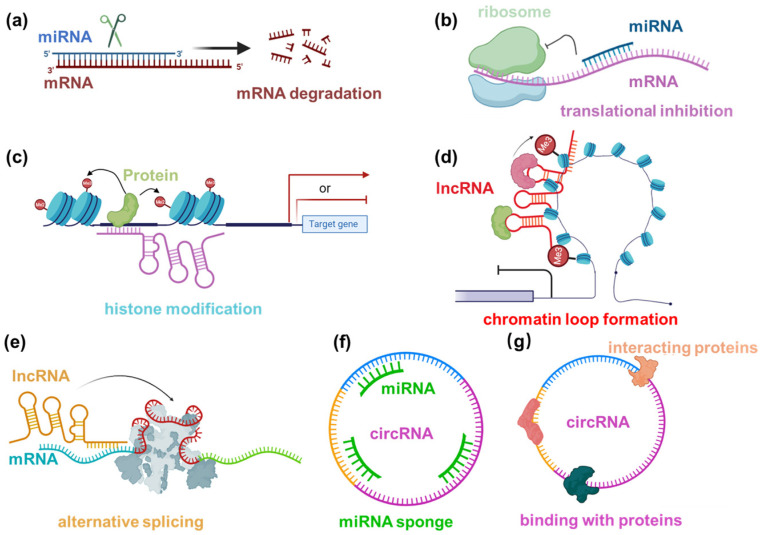
General mechanisms of different types of noncoding RNAs. The annotations for the symbols are in the same color in each panel. (**a**) mRNA degradation by miRNA; (**b**) translational inhibition by miRNA; (**c**) histone modification by lncRNA; (**d**) chromatin loop formation by lncRNA; (**e**) alternative splicing regulated by lncRNA; (**f**) circRNA serving as miRNA sponge; (**g**) circRNA interacting with proteins.

These mechanisms illustrate the diverse and intricate ways in which noncoding RNAs, including miRNAs, lncRNAs, and circRNAs, contribute to the regulation of plant development at the molecular level (Figure 1).

## 3. Noncoding RNAs Involved in Diverse Flowering Pathways

The regulation of flowering encompasses a complex network of interconnected pathways influenced by both internal and external factors. Early investigations in model plants unveiled several key pathways governing flowering time, including the photoperiodic, autonomous and vernalization, age, and GA pathways [40]. Among the pivotal regulators of flowering, noncoding RNAs have emerged as participants in various flowering pathways. Given that the roles of noncoding RNAs in the vernalization and autonomous pathways are closely intertwined, these two pathways will be merged and discussed in the following sections.

### 3.1. Photoperiodic Pathway

The photoperiodic pathway, characterized by the differential stability and activity of *CONSTANS* (*CO*), plays a pivotal role in modulating flowering time. *CO*, a rhythmically expressed gene, exhibits responsiveness to both circadian rhythms and light stimuli under varying daylengths [41]. This dynamic regulation influences the accumulation of FT, thereby contributing to the precise temporal control of flowering events. Noncoding RNAs play a crucial regulatory role in photoperiodic flowering by influencing circadian rhythms or upstream factors of CO. For instance, miR397b targets *CKB3*, impacting the activity of CCA1, a central component of the circadian clock. Overexpressing miR397b extends the circadian clock period, consequently delaying flowering. Conversely, miR397b is negatively regulated by CCA1, establishing a negative feedback loop of miR397b-*CKB3*-CCA1 [42]. The lncRNA *FLORE*, a natural antisense transcript of *CDF5*, inhibited the expression of *CDF5*. CDFs, under circadian transcriptional and post-translational control, are repressors of *CO* and *FT*; thus, overexpression of *FLORE* promotes flowering by upregulation of *CO* and *FT* [41,43]. The reciprocal inhibition of *FLORE* and *CDF5* is crucial for maintaining their oscillations [44]. 

Besides functioning upstream of *CO*, evidence also shows that noncoding RNAs function downstream of *CO* to regulate flowering. Overexpression of *RIFLA*, an lncRNA transcribed from the intron of *OsMADS56*, results in earlier flowering than the wild type under long-day conditions [45]. *OsMADS56* is the ortholog of the *Arabidopsis SOC1* in rice, positioned downstream of *CO*, facilitating the integration of signals conveyed by CO to promote flowering in plants. However, in contrast to facultative long-day plants, overexpression of *OsMADS56* in short-day crop plants like rice delays flowering under long-day conditions. Additionally, miR5200 in *Brachypodium distachyon* is epigenetically regulated by daylength and short-day induction, leading to delayed flowering by targeting *FTL1* and *FTL2* [46]. However, the diurnal or circadian expression patterns of these genes have not been elucidated. Understanding their expression patterns would significantly contribute to our comprehension of their roles within the photoperiodic pathway, allowing for better utilization and manipulation of their functions.

### 3.2. Autonomous Pathway and Vernalization Pathway

Plants require exposure to low temperatures to induce flowering, a phenomenon known as vernalization. Vernalization induces the inactivation of *FLC*, thereby promoting flowering. The autonomous pathway comprises the complex of *FLOWERING LOCUS CA* (*FCA*), *FLOWERING LOCUS PA* (*FPA*), *FLOWERING LOCUS KH DOMAIN* (*FLK*), and *FLOWERING LOCUS Y* (*FY*), responsible for repressing the expression of *FLC* by modifying 3′ end processing and antisense RNA polyadenylation at *FLC* [47]. If the complex of the autonomous pathway is absent, even vernalized plants cannot flower early. Hence, *FLC* occupies a central position between the vernalization and autonomous pathways [48]. LncRNAs such as *COOLAIR*, *COLDAIR*, and *COLDWRAP* are induced by cold treatment, mimicking winter conditions, and repress *FLC* expression. *COOLAIR*, an antisense lncRNA originating from the 3′ end of *FLC*, promotes flowering by repressing *FLC* expression [49,50]. *COLDAIR* is a sense lncRNA from the intron of *FLC*, and perturbating the association between *COLDAIR* and *FLC* delays flowering time in *Arabidopsis* [51]. Another lncRNA, *COLDWRAP*, derived from the promoter of *FLC*, also contributes to the de-repression of flowering in coordination with *COLDAIR* [52]. In addition, FPA, FCA, and FY can also regulate the alternative splicing of *COOLAIR*, recruiting FLD for H3K4me2 demethylation on *FLC*. These findings suggest that FLC is intricately regulated by noncoding RNAs, involving both the vernalization and autonomous pathways [47,53]. However, the interplay between noncoding RNAs such as *COOLAIR*, *COLDAIR*, and *COLDWRAP* in coordinating *FLC* modifications and regulating *FLC* expression requires further in-depth investigation. Additionally, datasets have shown that the flowering repressor genes *FLC* and *FLM* in Arabidopsis produce circRNAs [34]. These findings indicate that *FLC*, positioned at the intersection of the vernalization and autonomous pathways, is subject to regulation by various noncoding RNAs.

Besides the *FLC* locus, long noncoding RNAs generated from other loci have also been reported to regulate the vernalization pathway. A natural antisense transcript (NAT) from *MADS AFFECTING FLOWERING 4* (*MAF4*) called *MAS* is also cold-induced and activates *MAF4* expression to inhibit premature flowering [54]. Although the function of MAF4 appears to oppose the long noncoding RNAs generated from the *FLC* locus, this ensures that the plant flowers only after experiencing a sufficiently long period of low temperatures. Additionally, there are other long noncoding RNAs that have been identified through transcriptome data screening. LncRNA *TCONS_00035129* in *Brassica rapa* is induced by vernalization, positively correlating with *BraZF-HD21* [55]. LncRNA *AGL15X2* in *Beta vulgaris* L. reaches peak expression after 16 weeks of vernalization, promoting flowering by repressing *BvFT1* [56]. An intergenic lncRNA, *FLINC* from Arabidopsis, has been identified to play a role in temperature-mediated flowering. Overexpression of *FLINC* increases sensitivity to changes in ambient temperature [57]. However, how these long noncoding RNAs respond to vernalization and are induced for expression and the mechanistic understanding of these long noncoding RNAs remain incomplete, necessitating further molecular biology and genetic research for elucidation.

### 3.3. Aging Pathway

Plant age is a critical factor in regulating the transition to flowering, and research has indicated a close correlation between age and miR156/miR172. MiR156 exhibits higher expression levels in juvenility, gradually decreasing with age, crucial for maintaining the juvenile phase. Subsequent studies have demonstrated that the temporal expression of miR156 is regulated by sugars synthesized during photosynthesis. MiR156 targets the *SQUAMOSA promoter binding protein-like* (*SPL*) family, known as flowering-promoting factors, where the gradual downregulation of miR156 leads to a progressive increase in SPL expression. Overexpression of miR156 extends the juvenile stage and delays flowering in several plant species, including Arabidopsis, rice, *Zea mays* ssp. *mays* (maize), *Lycopersicon esculentum* (tomato), *Populus* × *canadensis* , *Lilium* × *formolongi*, and *Gossypium hirsutum* L. (cotton), underscoring the conserved function of this miRNA family [58,59,60,61,62]. Notably, in *Physcomitrella patens*, miR156 exhibits an opposing role by promoting the formation of leafy gametophores [63]. The SPL family encodes a group of transcription factors targeted by miR156. In Arabidopsis, miR156 targets 11 of 17 *SPL* members, with SPL3/4/5/9/15 being key contributors to flowering time regulation [64]. Gradually increased SPL9, 10, and 15 can bind to the promoter region of *MIR172B*, enhancing its expression and consequently downregulating the expression of the flowering repressor genes *APETALA2* (*AP2*) and *AP2-like*. Thus, miR172 acts downstream of miR156 and SPL9 and its abundance increases with plant age, exhibiting an expression pattern opposite to miR156. Overexpressing miR172 accelerates flowering in plants, contrasting the effects of miR156 [65]. Target analysis reveals that miR172 represses *AP2*, *SMZ*, *SNZ*, and *TOE1/2/3* [23,66,67]. Therefore, miR156-*SPLs*-miR172 defines an age pathway in a wide range of plants.

It is interesting that some other noncoding RNAs are predicted to target miR156 and miR172, acting as decoys to inhibit their binding to their respective target genes. For example, circ-CCR2, circ-SEC5A, and circ-EF1B may be involved in aging pathways, as they sponge miR156 or miR172 in soybean [38]. However, whether these circRNAs have an impact on aging and their actual role in regulating flowering time remains to be studied. Recently, heterologous expression of lncRNA *bra-miR156HG* from *Brassica campestris* (Chinese cabbage), which is assumed to be the precursor of miR156, leads to delayed flowering in Arabidopsis. However, overexpression of this lncRNA alters leaf morphology instead of changing flowering time in Chinese cabbage [68]. This indicates that various types of noncoding RNAs collaborate to regulate flowering in a single pathway, a phenomenon that is relatively scarce in plants currently.

Furthermore, age can also regulate miRNA splicing in a post-transcriptional manner. The splicing isoforms of miR528 are regulated by age. *Pri-miR528* has two splicing variants, *MIR528-T1* and *MIR528-T2*. In *MIR528-T1*, the 3′ end is shortened by 103 nucleotides due to proximal polyadenylation, while in *MIR528-T2*, it is shortened by 98 nucleotides due to intron splicing, although the mature sequence of miR528 is the same. Older plants tend to produce the *MIR528-T2* isoform, which weakens the ability to yield mature miR528, which promotes flowering by targeting *OsRFI2* [69]. This age-regulated splicing of miR528 represents a fine-tuning mechanism, enhancing its ability to promote flowering just before the reproductive transition. Further investigation is required to elucidate which factor regulates the splicing of miR528 and whether it is correlated with miR156 or miR172.

### 3.4. Phytohormone-Related Pathways

GA was initially considered the primary plant hormone regulating flowering, and its pathway has been extensively studied. DELLA proteins function as negative regulators in the GA signaling pathway, inhibiting the floral integrator factor *SOC1* and thereby impeding flowering. GA promotes the degradation of DELLA proteins, relieving SOC1 inhibition and promoting flowering [70]. MiR159 targets *MYB* transcription factors responsive to GA [71]. However, the inconsistencies in flowering phenotypes between miR159 and target *MYB* mutants suggest the involvement of other factors in this regulatory module [72]. 

Other plant hormone signaling components may indirectly regulate flowering by interacting with DELLA proteins. MiR390-*TAS3*-tasiRNAs participate in auxin-mediated flowering by targeting *auxin response factor* (*ARF*) *3/4* [73]. ARFs are negatively regulated by the auxin receptor Aux/IAA, which can indirectly modulate flowering by promoting GA20ox or inhibiting GA2ox to increase GA levels [74]. Additionally, Aux/IAA negatively regulates DELLA proteins, demonstrating the collaborative role of auxin and GA in flowering [75]. Similarly, OsmiR393 targets the auxin receptors *OsTIR1* and *OsAFB2*, and overexpression of miR393 affects auxin signaling, thereby impacting flowering time [76].

Brassinosteroids (BRs) are a group of polyhydroxylated steroidal hormones crucial in plant growth and development. Mutants lacking BRs exhibit delayed flowering, a phenotype restored by exogenous GA supplementation, demonstrating the synergistic action between BRs and GA [77]. Additionally, BR signaling can function independently of GA. In *Arabidopsis*, the BR signaling component BZR1 recruits the H3K27 demethylase ELF6 to the *FLC* gene locus, inducing H3K27 demethylation and consequently activating *FLC* expression and promoting flowering [78]. In rice, although there is no homologous gene to *FLC* [79], a study has indicated that overexpression of OsmiR397 enhances the plant’s BR signal, with an earlier heading time [12]. While the exact cause remains unclear, this suggests the potential existence of an alternative mechanism for BR-mediated flowering regulation in rice. 

Additionally, some noncoding RNA-mediated pathways regulating flowering remain unclear or challenging to categorize into specific pathways. For instance, in rice, overexpression of miR168 can elevate miR164 levels, delaying flowering [80]. MiR164 targets the transcription factors *CUC1* and *CUC2*, which are crucial for the formation of floral organ boundaries [81]. *Ef-cd* is an antisense lncRNA overlapping with *OsSOC1*, and it shortens the maturity duration, thereby accelerating heading in rice [82]. This example is challenging to categorize within the mentioned pathways since *SOC1* is a gene downstream in the process, where all pathways converge to trigger flowering. Recently, an intergenic lncRNA, *FLAIL*, was identified, and its mutants displayed an early flowering phenotype, indicating that *FLAIL* acts as a flowering repressor [30]. Mechanistic studies indicate that *FLAIL* reduces the expression of *LAC8* by influencing alternative splicing, consequently altering the flowering time. Interestingly, *LAC8* belongs to the laccase family along with the OsmiR397 target gene *OsLAC13*, hinting at potential flowering control pathways associated with laccase-related mechanisms in plants [12]. In citrus, overexpression of miR3954 promotes the production of phasiRNAs that target NAC genes, facilitating the regulation of flowering time [43]. 

In *Arabidopsis*, a circRNA derived from the intron of *At5g37720* significantly delays flowering time, with reduced expression of *FT* [58]. However, its mechanism of action remains unclear, potentially involving the circRNA binding to certain proteins and exerting trans-regulation over *FT* expression. Recently, the loss of function of miR394a and miR394b in Arabidopsis resulted in early flowering with reduced branching and lower seed production [41]. However, the phenotypic alteration in flowering time does not correlate with the known target gene *LCR*. Therefore, the mechanism by which miR394 regulates flowering remains unknown. Further in-depth research in the future will help us comprehend these unknown regulatory mechanisms. It may establish connections with known pathways or create novel pathways to explain these phenomena.

**Figure 2:**
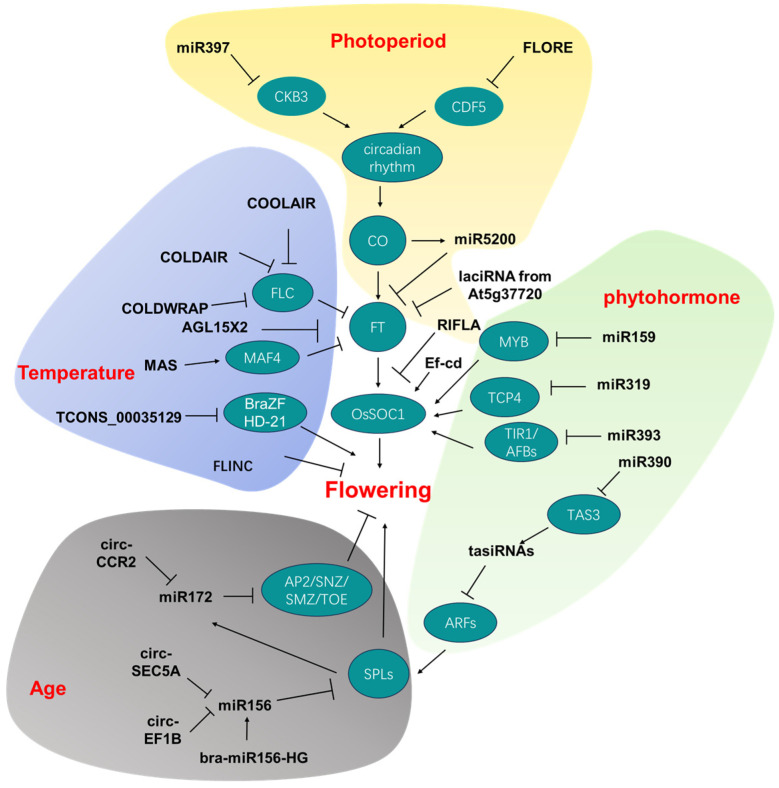
Noncoding RNAs involved in flowering time and their targets/downstream genes. Different colors of background represent different pathways, indicated by the word in red. *CKB3: Casein Kinase Beta subunit 3; CDF5: CYCLING DOF FACTOR 5; CO: CONSTANS; FT: FLOWERING LOCUS T; COOLAIR: COLD INDUCED LONG ANTISENSE INTRAGENIC RNA; COLDAIR: COLD-ASSISTED INTRONIC NONCODING RNA; FLORE: CDF5 LONG NONCODING RNA; FLC: FLOWERING LOCUS C; MAF4: MADS AFFECTING FLOWERING 4; BraZF-HD21:Bra026812; MYB: v-myb avian myeloblastosis viral oncogene homolog transcription factor; TCP: TEOSINTE BRANCHED 1, CYCLOIDEA, POLIFERATING CELL FACTORS; TIR1:TRANSPORT INHIBITOR RESISTANT1; AFB:AUXIN SIGNALING F-BOX; TAS3: Trans-Acting Short Interference RNA3; ARF: auxin response factor; SPL: SQUAMOSA promoter binding protein-like; AP2: APETALA2 transcription factors; SNZ: SCHNARCHZAPFEN; SMZ: SCHLAFMUTZE;TOE: TARGET OF EAT*.

In summary, noncoding RNAs intricately modulate plant flowering time through the complex web of pathways described above (Figure 2), although the mechanisms of some newly identified flowering-related noncoding RNAs are yet to be fully explored. The multifaceted involvement of noncoding RNAs underscores their pivotal role in the regulation of flowering time across various plant species (Table 1).

## 4. A Glimpse of Noncoding RNA in Flowering Adaption

Throughout the course of evolution and domestication, plant species have undergone adaptations to acclimate to changing environmental conditions. Noncoding RNAs, too, have experienced evolutionary selection to fine-tune the timing of flowering in plants. This scenario might occur within the regulatory regions of noncoding RNAs, where sequence variations induce subtle adjustments in noncoding RNA expression. For instance, in the upstream regulatory region of *MIR528*, two alleles, *MIR528-A1* and *MIR528-A2*, have been identified, predominantly found in indica and japonica rice, respectively. *MIR528-A2* exhibits a fragment deletion upstream of the transcription initiation site, leading to reduced expression levels [69]. This adaptive variation likely buffers some of the functions of miR528, allowing plants to adapt to higher latitudes and longer-daylight growth environments and suggests that during the expansion of rice cultivation northward, selective preservation of variations in the regulatory region of miR528 has occurred. 

Alternatively, such adaptations could arise within the mature or functional regions of noncoding RNAs, resulting in the emergence or disappearance of target genes. In a subsequent study, miR397 was shown to regulate flowering time in Arabidopsis by targeting the *CKB3*. However, the targeting of miR397-*CKB3* is exclusive to Arabidopsis. A G-to-U mutation at the 13th position of the mature miR397 sequence results in a robust match with *CKB3*. This mutation enhances plant fitness [42].

Plants evolved from unicellular algae, progressing through mosses (land plants), ferns (vascular plants), gymnosperms, and finally culminating in the appearance of angiosperms (flowering plants). Some miRNAs were already present before the emergence of flowering plants. For example, miR156 and miR390 emerged in land plants, while miR168 and miR397 emerged in vascular plants [90]. These facts raise questions such as how did they acquire the function of regulating flowering? Does this come with the generation of new target genes and the loss of old ones? These scenarios may reflect the Red Queen hypothesis from a noncoding RNA perspective [91]. Recent studies have even tested this hypothesis using de novo miRNA genes in two *Drosophila* species, suggesting that the hypothesis can be applied to noncoding RNAs [92].

Taken together, these discoveries emphasize the roles of noncoding RNAs in the environmental adaptation of flowering during the process of evolution. Future research efforts may focus on the effects of variations in other noncoding RNAs, which are less conserved than miRNAs and therefore exhibit a higher frequency of single nucleotide polymorphisms (SNPs) [93].

## 5. Strategies for Noncoding RNA Manipulation in Crop Flowering Control

To meet the ever-increasing demand for food production in a changing climate, the precise control of flowering time in crop plants is of paramount importance. A timely floral transition not only influences the seed quality and yield but also provides a buffer against environmental challenges such as pests and pathogens. Given the significant role noncoding RNAs play in regulating flowering time, manipulating their expression has become a promising avenue for crop breeders. In this section, we will explore various strategies for fine-tuning noncoding RNA expression in crop plants to optimize their flowering and enhance agricultural productivity.

The approaches for manipulating noncoding RNA expression vary depending on the specific type of noncoding RNA. In the case of miRNAs, the construction of mutant lines using short tandem target mimic (STTM) or target-resistant genes is a valuable means of studying miRNA functions [80,94]. For lncRNAs, RNA interference (RNAi) is a conventional technique for generating knock-down mutants that involves double-stranded small RNA molecules that target and silence specific genes, reducing their expression [95]. Genome editing techniques provide novel tools for modifying genes and creating new alleles. When it comes to circRNAs, strategies for studying their function have been documented [96], although the procedures differ somewhat from those used for linear genes. CRISPR/Cas9 technology is typically employed to introduce small insertions or deletions (InDels) into the target genome DNA, which may not substantially affect lncRNA functions. In such cases, multiple targets should be selected to facilitate substantial fragment deletions. CRISPR target selection is restricted by protospacer adjacent motif (PAM), typically a three-nucleotide sequence located immediately downstream of the guide RNA pairing site. The specific sequence of the PAM is determined by the type of Cas protein used in the CRISPR system [97]. Noncoding RNAs have limited options for target selection due to their relatively short length compared to protein-coding genes. Ongoing improvements in CRISPR/Cas9 tools for plant research allow for the expansion of the PAM repertoire and the creation of the desired edits [98,99,100,101]. By leveraging these genetic techniques, scientists can effectively modulate the flowering time of crop plants, resulting in economic benefits for farmers.

## 6. Conclusions and Perspective

In summary, the ongoing discoveries related to the identification, functional verification, and molecular actions of noncoding RNAs in plants have the potential to provide novel insights into flowering time regulation. This field holds promise as a molecular target for future crop breeding endeavors, with the potential to revolutionize agricultural practices and improve crop yields in an ever-changing world.

The rapid advancements in high-throughput sequencing technologies have significantly contributed to the identification of various noncoding RNAs critical for plant growth, development, and stress responses. As we have explored in this article, noncoding RNAs play pivotal regulatory roles in flowering time, with mechanisms that vary depending on their specific types. However, several challenges remain to be addressed. The functions of certain classes of RNAs, such as snoRNAs, snRNAs, and tRNA-derived small RNAs (tsRNAs) [102], in flowering time have yet to be elucidated, likely due to a lack of attention and established research strategies in these areas. Additionally, the complete understanding of the downstream factors and regulatory pathways associated with noncoding RNAs remains incomplete, potentially limiting their broader application in agriculture. To overcome these challenges, the integration of multi-omics data, including transcriptomes, epigenomes, and proteomes, in combination with pull-down experiments, may provide valuable insights into identifying interacting partners among DNA, RNA, and proteins.

Moreover, it is imperative to investigate the expression patterns and subcellular localization of noncoding RNAs, as this can offer crucial information for researchers to fine-tune the expression of noncoding RNAs at specific stages or in particular tissues or organelles. This targeted approach could enable the specific modulation of flowering while minimizing adverse effects on other developmental processes. The dynamic states of noncoding RNAs are also of significance, as many long noncoding RNAs have been shown to mediate phase separation in mammals [103], a physical state transition where certain components within a cell can form liquid droplets, segregating these components from the rest of the substances in a homogeneous system, which is involved in various biological processes [104]. However, the exploration of the relationship between phase separation and noncoding RNAs in plants lags behind that in animals. Although reports indicate that miRNAs are involved in phase separation [105], it remains largely unknown whether noncoding RNAs form droplets or condensates during their functions in plants.

## Figures and Tables

**Table 1:** Summary of noncoding RNAs in regulating flowering time.

Species	Categories	Names	Targets	Functions	Reference
*Arabidopsis*/rice/maize/tomato, etc.	miRNA	miR156	*SPLs*	delays flowering	[59,60]
*Arabidopsis*	miRNA	miR172	*AP2/SMZ/SNZ /TOE*	accelerates flowering	[83]
rice	miRNA	miR528	*OsRFI2*	promotes flowering under long-day conditions	[69]
*Arabidopsis*	miRNA	miR159	*MYB*	delays flowering in short day	[84]
*Arabidopsis*	miRNA	miR319	*TCP*	delays flowering in short day	[85]
*B. distachyon*	miRNA	miR5200	*FT*	promotes flowering under short-day conditions and inhibits flowering under long-day conditions	[86]
rice	miRNA	miR393	*OsTIR1/OsAFB2*	increases tiller number and promotes heading	[76]
rice	miRNA	miR168	*AGO1*	suppression of miR168 shortens flowering time	[80]
*Arabidopsis*	miRNA	miR397	*CKB3*	delays flowering time by modulating the circadian clock	[42]
*Arabidopsis*	miRNA	miR394a/ miR394b	*LCR*	loss-of-function showed early flowering with decreased branching and lower seed production	[87]
*Arabidopsis*	miRNA	miR390	*ARF3/ARF4*	delays flowering time	[63]
citrus	miRNA	miR3954	*NAC*	facilitates flowering time	[7]
*Arabidopsis*	miRNA	miR169	*NF-YA*	promotes early flowering through the abiotic stress response	[88]
*Arabidopsis*	miRNA	miR399	*PHO2*	promotes flowering at normal temperature	[89]
*Arabidopsis*	lncRNA	*COOLAIR*	*FLC*	promotes flowering	[8]
*Arabidopsis*	lncRNA	*COLDAIR*	*FLC*	promotes flowering	[51]
*Arabidopsis*	lncRNA	*COLDWRAP*	*FLC*	promotes flowering	[52]
*B. rapa*	lncRNA	*TCONS_00035129*	*BraZF-HD21*	promotes flowering	[55]
sugar beet	lncRNA	*AGL15X2*	*BvFT1*	promotes reproductive growth upon vernalization	[56]
*Arabidopsis*	lncRNA	*FLINC*	not clear	regulates temperature-mediated flowering	[57]
*Arabidopsis*	lncRNA	*FLORE*	*FT*	promotes flowering in a circadian-dependent manner	[44]
rice	lncRNA	*Ef-cd*	*OsSOC1*	shortens maturity duration with no yield penalty	[82]
*Arabidopsis*	lncRNA	*FLAIL*	*LAC8*	represses flowering	[30]
rice	lncRNA	*RIFLA*	*OsMADS56*	promotes flowering	[45]
Soybean	circRNA	circ-CCR2, circ-SEC5A, circ-EF1B	*miR172, miR156*	induces the floral meristem	[38]
Arabidopsis	circRNA	laciRNA from *At5g37720*	*FT*	delays flowering	[39]

## Data Availability

No new data were created or analyzed in this study. Data sharing is not applicable to this article.
